# Thermodynamic and Differential Entropy under a Change of Variables

**DOI:** 10.3390/e12030578

**Published:** 2010-03-16

**Authors:** Vladimir Hnizdo, Michael K. Gilson

**Affiliations:** 1 Health Effects Laboratory Division, National Institute for Occupational Safety and Health, Morgantown, West Virginia 26505, USA; 2 Center for Advanced Research in Biotechnology, University of Maryland Biotechnology Institute, Rockville, Maryland 20850, USA

**Keywords:** thermodynamic entropy, Shannon entropy, spatial entropy, non-Cartesian coordinates, canonical transformation, Jacobian

## Abstract

The differential Shannon entropy of information theory can change under a change of variables (coordinates), but the thermodynamic entropy of a physical system must be invariant under such a change. This difference is puzzling, because the Shannon and Gibbs entropies have the same functional form. We show that a canonical change of variables can, indeed, alter the spatial component of the thermodynamic entropy just as it alters the differential Shannon entropy. However, there is also a momentum part of the entropy, which turns out to undergo an equal and opposite change when the coordinates are transformed, so that the total thermodynamic entropy remains invariant. We furthermore show how one may correctly write the change in total entropy for an isothermal physical process in any set of spatial coordinates.

“Since no one understands what entropy is, by using this word you will have an advantage over your adversary in any debate.” John von Neumann’s advice to Claude Shannon, according to J. Campbell [1].

## 1. Introduction

The Gibbs entropy of classical statistical thermodynamics is, apart from some non-essential constants, the differential Shannon entropy [2] of the probability density function (pdf) in the phase space of the system under consideration. However, whereas the thermodynamic entropy is not expected to depend upon the choice of variables, the differential entropy can be changed by a transformation of variables. In particular, the differential entropy of a spatial pdf depends on the choice of the coordinates used to describe the spatial configuration of the system. Moreover, a change of variables can change not only the absolute differential entropy, but also its change on a change in the pdf, as shown below. This sensitivity to coordinates appears paradoxical, since a physically meaningful quantity ought to be independent of the choice of spatial coordinates. A similar concern was previously expressed in a critique of the concept of the differential entropy itself [3].

Here, we demonstrate that, for the thermodynamic entropy, a transformation of the spatial coordinates is accompanied by a compensating change of the entropy of the canonically conjugate momenta so that the full thermodynamic entropy remains invariant. The invariance of the full entropy stems from the fact that the Jacobian of a canonical transformation equals unity, and explicit demonstration of this invariance yields a simple formula for correcting a spatial entropy computed with transformed coordinates to yield correct full entropy changes. These results have application in calculations of spatial entropy from molecular simulations when Cartesian coordinates are transformed, for example to bond-angle-torsion [4] coordinates.

The paper is structured as follows. Section 2 reviews the formalism of entropy in classical statistical thermodynamics, defines a splitting of the full thermodynamic entropy into momentum and spatial parts for the case in which the spatial coordinates used are Cartesian, and shows that then the change in the spatial entropy equals the change in the total entropy, for an isothermal process. (Note that the spatial entropy of the solute part of a solute-solvent system is often termed the solute’s configurational entropy.) Sections 3 and 4 investigate the effects on the spatial and momentum entropy, respectively, of a transformation of the Cartesian coordinates to general spatial coordinates. Section 5, discusses how one may evaluate the change in the full thermodynamic entropy due to an isothermal process in terms of the change in the spatial entropy evaluated in non-Cartesian coordinates. Finally, Section 6 draws conclusions.

## 2. Spatial Entropy in Cartesian Coordinates

In classical statistical thermodynamics, the entropy *S* of a system described by coordinates *q*_1_, …, *q_s_* and the canonically conjugate momenta *p*_1_, …, *p_s_*, briefly *p* and *q*, respectively, is given in terms of the system’s pdf *ρ*(*p, q*) in the phase space (*p, q*) as [5]

(1) $$S=-k_{B}\int dp\int dq\rho (p,q)ln[h^{s}\rho (p,q)]$$

Here, *k_B_* is Boltzmann’s constant, and the factor *h^s^*, where *h* = 2*π*ħ is Planck’s constant (quasi-classically, the number of states in a volume element Δ*_p_*Δ*_q_* of the phase space (*p, q*) is Δ*_p_*Δ*_q_/h^s^*, see e.g., [6]), ensures that the argument of the logarithm is dimensionless. The probability distribution function *ρ*(*p, q*) is given by the Boltzmann-Gibbs distribution

(2) $$\rho (p,q)=\frac{1}{h^{s}Z}e^{-\beta E(p,q)}$$

where *β* = 1/(*k_B_T*), with *T* being the absolute temperature, *E*(*p, q*) is the system’s energy, and

(3) $$Z=\frac{1}{h^{s}}\int dp\int dqe^{-\beta E(p,q)}$$

is the partition function, which is the distribution’s normalization constant divided by *h^s^* to make it dimensionless. The entropy *S* can be written in terms of the partition function *Z* as

(4) $$S=\frac{1}{T}\langle E\rangle +k_{B}lnZ$$

where

(5) 〈E〉=∫dp∫dqρ(p,q)E(p,q)

is the mean (expectation) value of the energy *E*(*p, q*).

Let us assume that the coordinates *q* are Cartesian. Then the energy *E*(*p, q*) is the sum of a kinetic energy, *K*, which is a function of the momenta *p* only, $K=\frac{1}{2}\sum_{i=1}^{s}p_{i}^{2}/m_{i}$, where *m_i_* are the masses associated with degrees of freedom *i*, and a potential energy *U* = *U*(*q*), which depends only on spatial coordinates. The probability distribution (2) and the partition function (3) now factorize as

(6) $$\rho (p,q)=\rho_{m}(p)\rho_{s}(q)$$

(7) $$Z=Z_{m}Z_{s}$$

where,

(8) $$\rho_{m}(p)=\frac{1}{h^{s}Z_{m}}e^{-\frac{1}{2}\beta }\sum_{i}p_{i}^{2}/m_{i}$$

(9) $$\rho_{s}(q)=\frac{1}{Z_{s}}e^{-\beta U(q)}$$

are momentum and spatial probability distributions with normalization constants

(10) $$Z_{m}=\frac{1}{h^{s}}\int dpe^{-\frac{1}{2}\beta \sum_{i}p_{i}^{2}/m_{i}}=\frac{1}{h^{s}}{\left(\frac{2\pi \bar{m}}{\beta }\right)}^{s/2},\bar{m}={\left(\prod_{i=1}^{s}m_{i}\right)}^{1/s}$$

(11) $$Z_{s}=\int dqe^{-\beta U(q)}$$

which can be termed, respectively, the momentum and spatial partition functions. It should be noted that neither of these partition functions are dimensionless; the treatment of the factor 1/*h^s^* is discussed later in this section.

Accordingly, the full entropy (1) can now be separated as

(12) $$S=S_{m}+S_{s}$$

where *S_m_* is a momentum entropy which can be evaluated in closed form,

(13) $$S_{m}=-k_{B}\int dp\rho_{m}(p)ln[h^{s}\rho_{m}(p)]=k_{B}\frac{s}{2}[1+ln(2\pi \bar{m}/\beta )]-k_{B}ln(h^{s})$$

and *S_s_* is a spatial entropy,

(14) $$S_{s}=-k_{B}\int dq\rho_{s}(q)ln[\rho_{s}(q)]$$

Similarly to (4), the spatial entropy *S_s_* can be written as

(15) $$S_{s}=\frac{1}{T}\langle U\rangle +k_{B}lnZ_{s}$$

where

(16) $$\langle U\rangle =\int dq\rho_{s}(q)U(q)$$

is the mean value of the potential energy *U*(*q*).

A factor of *h^s^* is included in the definition of the momentum entropy (13) so that the full entropy *S_m_* + *S_s_* has the correct physical dimensions of energy/temperature. The association of this factor with the momentum entropy is arbitrary (as was its inclusion in the momentum partition function (10)); it could instead have been included in the spatial entropy. Unfortunately, the factor cannot be split so that both parts of the full entropy are dimensionless. Thus, neither of the “partial” entropies *S_m_* and *S_s_* is correctly dimensioned. Nonetheless, the troubling dimensions cancel for differences in *S_m_* and *S_s_* arising from changes in the pdf, since the relevant terms appear in the arguments of logarithms, and so such differences are physically meaningful.

It is evident from Equation (13) that the momentum entropy does not depend upon the spatial pdf *ρ_s_*(*q*), but only upon the momentum pdf *ρ_m_*(*p*), which in turn depends only on the atomic masses and the temperature. As a consequence, for an isothermal physical process with a fixed set of atoms, the change in total entropy equals the change in spatial entropy:

(17) $$\Delta S=\Delta S_{s}(\Delta T=0)$$

More generally, this equation holds in any coordinate system for which the kinetic energy *K* is independent of the spatial coordinates, *K* = *K*(*p*).

Note that the spatial entropy defined here is akin to the configurational entropy. However, the latter usually refers specifically to the entropy associated with the conformational fluctuations of a molecule in solution, and is therefore exclusive of the solvent entropy [9]. The spatial entropy is more general, as it may refer to the whole system or any of its parts.

## 3. Spatial Entropy under a Coordinate Transformation

It is sometimes of interest to compute the change in total entropy, Δ*S*, associated with an isothermal molecular process, such as protein-ligand binding or protein-folding. Equation (17) shows that the change in total entropy can be obtained by computing the change in the spatial entropy in Cartesian coordinates. However, Cartesian coordinates are not always optimal for this purpose, because the pdf in Cartesian coordinates includes many coordinate dependencies that are rather easily removed by transforming to more natural coordinates, such as bond-angle-torsion (BAT) coordinates [4]. For example, even the Cartesian coordinates of a single atom, (*x_i_, y_i_, z_i_*), can be strongly correlated with each other, due to the natural tendency of each atom to move in a circular trajectory corresponding to a bond-rotation. This motion is readily captured by a single torsional variable. For this reason, a transformation from Cartesian coordinates to suitably defined internal coordinates of the molecular system under consideration, plus the coordinates of the translation and rotation of the system as a whole, is often performed.

Transforming from Cartesian coordinates *q* to new coordinates *Q*(*q*) (with an inverse *q* = *q*(*Q*)), transforms the probability density function (pdf) *ρ_s_*(*q*) into a pdf *ρ̃_s_*(*Q*) of the new coordinates *Q* according to a rule of general probability theory as

(18) $${\tilde{\rho }}_{s}(Q)=\rho_{s}[q(Q)]J(Q)=\frac{J(Q)}{Z_{s}}e^{-\beta U(Q)}$$

where *J*(*Q*) is the Jacobian of the transformation. The differential Shannon entropy *H_q_* of information theory, defined as [2]

(19) $$H_{q}=-\int dq\rho_{s}(q)ln[\rho_{s}(q)]$$

can be written in terms of the new coordinates as

(20) $$H_{q}=-\int dQJ(Q)\rho_{s}[q(Q)ln\{\rho_{s}[q(Q)]\}=-\int dQ{\tilde{\rho }}_{s}(Q)ln[{\tilde{\rho }}_{s}(Q)/J(Q)]=H_{Q}+\langle lnJ(Q)\rangle$$

where

(21) $$H_{Q}=-\int dQ{\tilde{\rho }}_{s}(Q)ln[{\tilde{\rho }}_{s}(Q)]$$

is the Shannon entropy of the transformed pdf *ρ̃_s_*(*Q*) and

(22) $$\langle lnJ(Q)\rangle =\int dQ{\tilde{\rho }}_{s}(Q)ln[J(Q)]$$

is the expectation value of the logarithm of the Jacobian *J*(*Q*).

Based upon Equation (20), the spatial part of the thermodynamic entropy (14) in Cartesian coordinates *q*, *S_s_* = *k_B_H_q_*, transforms to a different value on changing to internal coordinates *Q*:

(23) $${\tilde{S}}_{s}=S_{s}-k_{B}\langle lnJ(Q)\rangle$$

where *S̃_s_* = *k_B_H_Q_*. This result may seem troubling, since the thermodynamic entropy of a physical system should not depend on the choice of the spatial coordinates used. Nonetheless, this Jacobian correction is real and is not even guaranteed to cancel when one computes the difference between two entropies associated with a change in the potential function and a consequent change in the physical pdf from *ρ* to some *ρ′*. That is, the change Δ*S_s_* in the Cartesian spatial entropy *S_s_* arising from a change $\rho_{s}(q)\to \rho_{s}^{\prime }(q)$ in the pdf of *q* does not necessarily equal the change Δ*S̃_s_* in the transformed spatial entropy *S̃_s_* arising from the corresponding change ${\tilde{\rho }}_{s}(Q)\to {\tilde{\rho }}_{s}^{\prime }(Q)$ in the transformed pdf of *Q*. This is because the Jacobian can vary with *Q*, so its contribution to the entropy for *ρ* (22) can differ from that for *ρ′*

(24) $$\langle lnJ(Q)\rangle \prime =\int dQ{\tilde{\rho }}_{s}^{\prime }(Q)ln[J(Q)]$$

Hence,

(25) $$\Delta S_{s}=S_{s}^{\prime }-S_{s}={\tilde{S}}_{s}^{\prime }+k_{B}{\langle lnJ(Q)\rangle }^{\prime }-{\tilde{S}}_{s}-k_{B}\langle lnJ(Q)\rangle \neq \Delta {\tilde{S}}_{s}$$

We note that a similar treatment of the transformation of spatial entropy under a change of coordinates has been given in [7].

## 4. Momentum Entropy under a Coordinate Transformation

The coordinate transformation *q* → *Q* = *Q*(*q*) is called a point transformation because the new coordinates *Q* are functions only of the old coordinates *q*; that is, they do not involve the old conjugate momenta *p*. (A general point transformation may also involve an explicit dependence on time.) A point transformation of the spatial coordinates is associated with a canonical transformation of the full phase space coordinate system; *i.e.*, of both the spatial and momentum coordinates, such that *p, q* → *P* = *P*_(*p, q*)_, *Q* = *Q*_(*q*)_, with the inverses *p* = *p*(*P,Q*), *q* = *q*(*Q*). In a canonical transformation, the new variables *P,Q* remain canonically conjugate, which means that

(26) $$P_{i}=\frac{\partial L}{\partial {\dot{Q}}_{i}},i=1,\ldots ,s$$

where *L* = *L*(*Q, Q̇, t*), with *Q̇* = *dQ/dt*, is the system’s Lagrangian expressed in terms of *Q*. Classical statistical thermodynamics is based on the Hamilton formalism of mechanics, and property (26) ensures that the Hamilton equations in terms of *P, Q* retain their canonical form. Here, an important property of a canonical transformation *p, q* → *P,Q* is that its Jacobian equals unity [8]. This means that the phase-space volume element of an integration in the full phase space is invariant:

(27) dpdq=dPdQ

A point transformation *q* → *Q* = *Q*(*q*) itself has in general a Jacobian *J*(*Q*) ≠ = 1, and so *dq* = *J*(*Q*) *dQ*. Thus, for invariance (27) to hold, the momentum volume element *dp* must transform in a canonical transformation as

(28) $$dp=\frac{1}{J(Q)}dP$$

Using (27), we can write the full entropy (1) in terms of an integral in the new phase space variables *P,Q* simply as

(29) $$S=-k_{B}\int dP\int dQ\tilde{\rho }(P,Q)ln[h^{s}\tilde{\rho }(P,Q)]$$

where

(30) $$\tilde{\rho }(P,Q)=\rho (p(P,Q),q(Q))=\frac{1}{h^{s}Z}e^{-\beta [K(P,Q)+U(Q)]}$$

is the pdf of the new canonical variables *P,Q*. Note that because in general *p* = *p*(*P,Q*), the kinetic energy *K* is now a function of not only the momenta *P*, but also of the non-Cartesian coordinates *Q*: *K* = *K*(*P,Q*). Like any joint pdf, (30) may be factorized by means of the product rule as

(31) $$\tilde{\rho }(P,Q)={\tilde{\rho }}_{m}(P|Q){\tilde{\rho }}_{s}(Q)$$

where

(32) $${\tilde{\rho }}_{s}(Q)=\int dP\tilde{\rho }(P,Q)$$

is the marginal pdf of the coordinates *Q*, and

(33) $${\tilde{\rho }}_{m}(P|Q)=\frac{\tilde{\rho }(P,Q)}{{\tilde{\rho }}_{s}(Q)}$$

is the conditional pdf of *P* given *Q*. Using (30), (28) and (10), it can be verified that the marginal pdf (32) equals the spatial probability density (18) that was introduced in Sec. 2 under the same symbol:

(34) $${\tilde{\rho }}_{s}(Q)=\frac{1}{h^{s}Z}e^{-\beta U(Q)}\int dPe^{-\beta K(P,Q)}=\frac{J(Q)}{h^{s}Z}e^{-\beta U(Q)}\int dpe^{-\beta K(p)}=\frac{J(Q)}{Z}e^{-\beta U(Q)}Z_{m}$$

Here, the transformation *dP* = *J*(*Q*) *dp* was performed, which reverts the kinetic energy *K*(*P,Q*) to being a function of the Cartesian momenta *p* only, *K* = *K*(*p*), since this transformation is the inverse of that which made the kinetic energy *K*(*p*) a function of both *P* and *Q*. (The transformation *P* → *p* = *p*(*P,Q*) has an inverse *P* = *P*(*p,Q*) that is such that *K*(*P*(*p,Q*), *Q*) is in fact a function of *p* only.) Since *Z_m_/Z* = 1/*Z_s_* see Equation (7)], the last line of (34) indeed yields the spatial pdf (18).

Using (31) and the fact that, like any conditional pdf, the distribution (33) is properly normalized (*i.e.*, ∫*dP* *ρ̃_m_*(*P|Q*) = 1 at any *Q*), the full entropy (29) can be written as

(35) $$S=-k_{B}\int dP\int dQ{\tilde{\rho }}_{m}(P|Q){\tilde{\rho }}_{s}(Q)ln[h^{s}{\tilde{\rho }}_{m}(P|Q)]-k_{B}\int dP\int dQ{\tilde{\rho }}_{m}(P|Q){\tilde{\rho }}_{s}(Q)ln[{\tilde{\rho }}_{s}(Q)]=k_{B}\langle H_{P}(Q)\rangle -k_{B}ln(h^{s})+k_{B}H_{Q}$$

where

(36) $$\langle H_{P}(Q)\rangle =\int dQ{\tilde{\rho }}_{s}(Q)H_{P}(Q)$$

is the mean (expectation) value with respect to *Q* of the Shannon entropy of the conditional pdf *ρ̃_m_*(*P|Q*),

(37) $$H_{P}(Q)=-\int dP{\tilde{\rho }}_{m}(P|Q)ln[{\tilde{\rho }}_{m}(P|Q)]$$

and *H_Q_* is the Shannon entropy (21) of *ρ̃_s_*(*Q*). In analogy with (12), Equation (35) in turn can be written as

(38) $$S={\tilde{S}}_{m}+{\tilde{S}}_{s}$$

where

(39) $${\tilde{S}}_{m}=k_{B}\langle H_{P}(Q)\rangle -k_{B}ln(h^{s})$$

may be termed the momentum entropy associated with the new momenta *P*, and

(40) $${\tilde{S}}_{s}=k_{B}H_{Q}$$

is the spatial entropy in the new spatial coordinates *Q*. Equations (12) and (38), together with (23), straightforwardly yield the transformation of the momentum entropy:

(41) $${\tilde{S}}_{m}=S_{m}+k_{B}\langle lnJ(Q)\rangle$$

This relation complements the transformation (23) of the spatial entropy so that, on a point transformation *q* → *Q* = *Q*(*q*), a change in the spatial entropy is compensated by a change in the momentum entropy and the full entropy remains invariant: *S_m_* + *S_s_* = *S̃_m_* + *S̃_s_*.

## 5. Total Entropy in Terms of the Spatial Entropy in Non-Cartesian Coordinates

It is now easy to show how the change in the spatial entropy of a molecular system, calculated using a non-Cartesian system of coordinates, can be corrected to provide the corresponding change in the full thermodynamic entropy of the system, which is the quantity of physical interest. We consider the entropy change arising from some physicochemical process which changes the phase-space pdf of the system from *ρ* to *ρ′*. The molecular species of interest is considered to be present at a standard concentration *C^∘^*, which corresponds to an isolated molecule in a container of volume *V^∘^* = 1/*C^∘^* [9]. For an isothermal process, and with Cartesian coordinates, the momentum pdf is unchanged, so that there is no change in the momentum entropy, *S_m_*. The change Δ*S* of the full thermodynamic entropy therefore equals the change in the Cartesian spatial entropy *S_s_*:

(42) $$\Delta S=\Delta (S_{m}+S_{s})=\Delta S_{s}(\Delta T=0)$$

If non-Cartesian coordinates *Q* are used, as in many methods for calculating the spatial entropy, a change Δ*S̃_s_* in the entropy of the coordinates *Q* obtained in this way can be corrected to the change Δ*S_s_* in the Cartesian spatial entropy *S_s_* using (23):

(43) $$\Delta S=\Delta S_{s}=\Delta {\tilde{S}}_{s}+k_{B}\Delta \langle lnJ(Q)\rangle$$

Here, the correction term

(44) Δ〈lnJ(Q)〉=〈lnJ(Q)〉′−〈lnJ(Q)〉

is the difference between the means, evaluated with the changed and original spatial distributions ${\tilde{\rho }}_{s}^{\prime }(Q)$ and *ρ̃_s_*(*Q*), respectively, of the logarithm of the Jacobian of the transformation *q* → *Q* = *Q*(*q*). If molecular simulations are used for the evaluation of spatial entropy, then 〈ln *J*(*Q*)〉 is evaluated easily as a simple arithmetic mean,

(45) $$\langle lnJ(Q)\rangle =\frac{1}{n}\sum_{i=1}^{n}lnJ(Q_{i})$$

where *Q_i_, i* = 1*, …, n* is a sample of the coordinates obtained from snapshots of the simulation trajectory.

We now consider the specific case where *Q* represents bond-angle-torsion (BAT) coordinates. For *N* atoms, *Q* comprises 3 external translational coordinates **r**_ex_ = (*x*_ex_, *y*_ex_, *z*_ex_), 3 external rotational coordinates *θ*_ex_, *ϕ*_ex_, *ψ*_ex_, and 3*N* − 6 internal coordinates of *N* − 1 bond lengths *b* = (*b*_2_, …, *b_N_*), *N* − 2 bond angles *θ* = (*θ*_3_, …, *θ_N_*), and *N* − 3 torsional angles *ϕ* = (*ϕ*_4_, …, *ϕ_N_*), where the subscripts indicate the atoms to which the internal coordinates correspond. The Jacobian for this transformation is given as [4]

(46) $$J_{\text{BAT}}=\text{sin}\theta_{ex}b_{2}^{2}\prod_{i=3}^{N}b_{i}^{2}\text{sin}\theta_{i}\equiv \text{sin}\theta_{ex}J(b,\theta )$$

and the spatial pdf in Cartesian coordinates *q* = *x*, *ρ_s_*(*x*), is thus transformed into the following pdf in BAT coordinates:

(47) $${\tilde{\rho }}_{s}(r_{ex},\theta_{ex},\phi_{ex},\psi_{ex},b,\theta ,\phi )=\rho_{s}(x(r_{ex},\theta_{ex},\phi_{ex},\psi_{ex},b,\theta ,\phi ))J_{\text{BAT}}=\frac{1}{Z_{s}}e^{-\beta U(b,\theta ,\phi )}\text{sin}\theta_{ex}J(b,\theta )$$

(48) $$Z_{s}=8\pi^{2}V^{\circ }\int \int \int dbd\theta d\phi e^{-\beta U(b,\theta ,\phi )}J(b,\theta )$$

where we have used expression (9) for *ρ_s_*, along with the fact that the potential energy *U*(*x*) of a molecule of *N* atoms that is not located in an external field depends only on its 3*N* − 6 internal BAT coordinates (*b, θ, ϕ*). The right-hand side of (47) may be written as a product of two factors, one depending on only the external coordinate *θ*_ex_ and the other on only the internal coordinates (*b, θ, ϕ*). Therefore, the joint pdf (47) can be factorized as

(49) $${\tilde{\rho }}_{s}(r_{ex},\theta_{ex},\phi_{ex},\psi_{ex},b,\theta ,\phi )={\tilde{\rho }}_{ex}(r_{ex},\theta_{ex},\phi_{ex},\psi_{ex}){\tilde{\rho }}_{in}(b,\theta ,\phi )$$

where

(50) $${\tilde{\rho }}_{ex}(r_{ex},\theta_{ex},\phi_{ex},\psi_{ex})=\frac{\text{sin}\theta_{ex}}{8\pi^{2}V^{\circ }}$$

and

(51) $${\tilde{\rho }}_{in}(b,\theta ,\phi )=\frac{8\pi^{2}V^{\circ }}{Z_{s}}e^{-\beta U(b,\theta ,\phi )}J(b,\theta )$$

Here, *ρ̃*_ex_, the marginal pdf of the external coordinates, is clearly normalized because

(52) $$\int_{V^{\circ }}dr_{ex}\int_{0}^{\pi }\text{sin}\theta_{ex}d\theta_{ex}\int_{0}^{2\pi }d\phi_{ex}\int_{0}^{2\pi }d\psi_{ex}=8\pi^{2}V^{\circ }$$

and *ρ̃*_in_(*b, θ, ϕ*), the marginal pdf of the internal BAT coordinates, is normalized based on the definition of *Z_s_* Equation(48)].

The entropy of the joint pdf (49) now separates as

(53) $${\tilde{S}}_{s}={\tilde{S}}_{ex}+{\tilde{S}}_{in}$$

where *S̃*_ex_ and *S̃*_in_ are the entropies of the marginal pdf’s *ρ̃*_ex_ and *ρ̃*_in_, respectively. Using expression (50) for *ρ̃*_ex_, the external entropy *S̃*_ex_ can be written as

(54) $${\tilde{S}}_{ex}=-k_{B}\int_{V^{\circ }}dr_{ex}\int_{0}^{\pi }d\theta_{ex}\int_{0}^{2\pi }d\phi_{ex}\int_{0}^{2\pi }d\psi_{ex}{\tilde{\rho }}_{ex}ln{\tilde{\rho }}_{ex}=k_{B}ln(8\pi^{2}V^{\circ })-k_{B}\int_{0}^{\pi }d\theta_{ex}\text{sin}\theta_{ex}ln(\text{sin}\theta_{ex})=k_{B}ln(8\pi^{2}V^{\circ })-k_{B}\langle ln(\text{sin}\theta_{ex})\rangle$$

where 〈ln(sin *θ*_ex_)〉 is the mean of ln(sin *θ*_ex_) for a uniform distribution of molecular orientations. Finally, using Equations (23), (46), (53) and (54), we have for the spatial entropy in Cartesian coordinates, *S_s_*:

(55) $$S_{s}={\tilde{S}}_{ex}+{\tilde{S}}_{in}+k_{B}\langle lnJ_{\text{BAT}}\rangle =k_{B}ln(8\pi^{2}V^{\circ })-k_{B}\langle ln(\text{sin}\theta_{ex})\rangle +{\tilde{S}}_{in}+k_{B}\langle ln[\text{sin}\theta_{ex}J(b,\theta )]\rangle =k_{B}ln(8\pi^{2}V^{\circ })+{\tilde{S}}_{in}+k_{B}\langle ln[J(b,\theta )]\rangle$$

In previous work [10], *S_s_* has been written in terms of the BAT coordinates (*b, θ, ϕ*) as

(56) $$S_{s}=k_{B}ln(8\pi^{2}V^{\circ })-k_{B}\int db\int d\theta \int d\phi J(b,\theta )\rho (b,\theta ,\phi )ln[\rho (b,\theta ,\phi )]$$

where *ρ*(*b, θ, ϕ*) is a distribution function of the internal BAT coordinates that becomes the normalized marginal pdf *ρ̃*_in_(*b, θ, ϕ*) on multiplication by *J*(*b, θ*),

(57) $$\rho (b,\theta ,\phi )=\frac{1}{J(b,\theta )}{\tilde{\rho }}_{in}(b,\theta ,\phi )$$

Expression (56) is entirely consistent with the presented formalism, as it can be rewritten in terms of the properly normalized marginal pdf *ρ̃*_in_(*b, θ, ϕ*) and *J*(*b, θ*) to give

(58) $$S_{s}=k_{B}ln(8\pi^{2}V^{\circ })-k_{B}\int db\int d\theta \int d\phi {\tilde{\rho }}_{in}(b,\theta ,\phi )ln[{\tilde{\rho }}_{in}(b,\theta ,\phi )]+k_{B}\int db\int d\theta \int d\phi {\tilde{\rho }}_{in}(b,\theta ,\phi )ln[J(b,\theta )]=k_{B}ln(8\pi^{2}V^{\circ })+{\tilde{S}}_{in}+k_{B}\langle ln[J(b,\theta )]\rangle$$

which is identical with (55). The results of [10] and other work that adopted a similar evaluation of *S_s_* as that in (56) are thus not affected by our findings.

The approaches outlined above for evaluating the spatial entropy in Cartesian coordinates in BAT coordinates avoid potential shortcomings that may arise from approximating *J*(*b, θ*) as a constant equal to its value at the equilibrium values *b* = *b*_0_ and *θ* = *θ*_0_ of the internal BAT coordinates. In particular, although most of these coordinates are “hard” and therefore make nearly constant contributions to the Jacobian, this is by no means the case for the pseudo-bond and pseudo-angles often used to define the position and orientation of one molecule relative to another in a noncovalent complex [11].

One circumstance in which the Jacobian can be approximated as constant is that in which the molecule occupies only a single, reasonably narrow energy well with its local minimum at internal coordinates *b*_0_, *θ*_0_, *ϕ*_0_. One may then make the approximation *J*(*b, θ*) ≈ *J*(*b*_0_, *θ*_0_), in which case the pdf of Equation (51) simplifies to

(59) $${\tilde{\rho }}_{in}(b,\theta ,\phi )\approx \frac{e^{-\beta U(b,\theta ,\phi )}}{\int \int \int dbd\theta d\phi e^{-\beta U(b,\theta ,\phi )}}$$

If, as a further approximation, the harmonic approximation is used for the potential energy *U*(*b, θ, ϕ*), this pdf becomes a multivariate normal (Gaussian) distribution, the entropy of which can be evaluated in closed form, yielding for the entropy of internal coordinates the following estimate:

(60) $${\tilde{S}}_{in}\approx \frac{k_{B}}{2}(3N-6)+\frac{k_{B}}{2}ln\frac{{\left(2\pi /\beta \right)}^{3N-6}}{\text{det}F(b_{0},\theta_{0},\phi_{0})}$$

where *F*(*b*_0_, *θ*_0_, *ϕ*_0_) is the Hessian matrix of the harmonic potential energy at the energy minimum. The widely used quasiharmonic approximation for estimation of the configurational entropy from molecular simulations was based in its original formulation [12] on the assumption that *F* ≈ ∑^−1/β^ where ∑ is the covariance matrix of a simulation sample of internal coordinates. Using now Equations (55) and (60), the spatial entropy in Cartesian coordinates is obtained as

(61) $$S_{s}\approx k_{B}ln(8\pi^{2}V^{\circ })+\frac{k_{B}}{2}(3N-6)+\frac{k_{B}}{2}ln\frac{{\left(2\pi /\beta \right)}^{3N-6}}{\text{det}F(b_{0},\theta_{0},\phi_{0})}+k_{B}ln[J(b_{0},\theta_{0})]$$

With these approximations, an isothermal process that changes the conformation of the system produces a change Δ*S_s_* in the spatial entropy *S_s_* given by

(62) $$\Delta S_{s}=S_{s}^{\prime }-S_{s}\approx \frac{k_{B}}{2}ln\frac{\text{det}F(b_{0},\theta_{0},\phi_{0})}{\text{det}F(b_{0}^{\prime },\theta_{0}^{\prime },\phi_{0}^{\prime })}+k_{B}ln\frac{J(b_{0}^{\prime },\theta_{0}^{\prime })}{J(b_{0},\theta_{0})}$$

where the primed quantities pertain to the changed system. Clearly, the Jacobian-dependent term may be neglected only when the equilibrium Jacobians of the two conformations are approximately the same. As noted above, this approximation holds to good accuracy when $b_{0}^{\prime }\approx b_{0}$ and $\theta_{0}^{\prime }\approx \theta_{0}$

Another perspective on this condition is also of interest. We first note that $J_{\text{BAT}}^{2}=\text{det}{𝒢}^{-1}/\prod_{i=1}^{N}m_{i}^{3}$, where 𝒢 is the total kinetic-energy matrix in the BAT coordinates, and *m_i_* are the masses of the atoms; 𝒢, like *J*_BAT_, is a function of the BAT coordinates. This expression follows from the fact that 𝒢 = ℬℳ^−1^ℬ^T^, where ℳ = diag(*m*_1_, *m*_1_, *m*_1_, …, *m_N_,m_N_,m_N_*) and ℬ is a 3*N* × 3*N* matrix whose elements are the partial derivatives *∂Q_i_/∂x_j_* of all the BAT coordinates with respect to the Cartesian coordinates, so that det ℬ^−1^ = *J*_BAT_ (see, e.g., [14]). Using this identity, along with, e.g., Equations (2.34) and (3.5) of [13], one may show that

(63) $$J(b,\theta )={\left(\frac{\sum_{i=1}^{N}m_{i}}{\prod_{i=1}^{N}m_{i}}\right)}^{3/2}\sqrt{\frac{\text{det}I(b,\theta ,\phi )}{\text{det}G(b,\theta ,\phi )}}$$

where *I*(*b, θ, ϕ*) is the matrix of the instantaneous inertia tensor and *G*(*b, θ, ϕ*) is the kinetic-energy matrix of the 3*N* − 6 internal degrees of freedom. Note that det *I* = *I*_1_*I*_2_*I*_3_, where *I_i_* are the principal moments of inertia. The Jacobian term in (62) therefore may be written as

(64) $$k_{B}ln\frac{J(b_{0}^{\prime },\theta_{0}^{\prime })}{J(b_{0},\theta_{0})}=\frac{k_{B}}{2}ln[\frac{\text{det}I(b_{0}^{\prime },\theta_{0}^{\prime },\phi_{0}^{\prime })}{\text{det}I(b_{0},\theta_{0},\phi_{0})}\frac{\text{det}G(b_{0},\theta_{0},\phi_{0})}{\text{det}G(b_{0}^{\prime },\theta_{0}^{\prime },\phi_{0}^{\prime })}]$$

## 6. Conclusions

We have addressed the situation in which one wishes to compute Δ*S* for a classically treated, isothermal physical process where the phase-space probability distribution *ρ*(*p, q*) goes to *ρ′*(*p, q*). In the biophysical context, this might be a binding or folding process. If (*p, q*) are Cartesian, then we can factorize *ρ*(*p, q*) as *ρ_m_*(*p*)*ρ*_*s*_(*q*) and accordingly decompose the entropy *S* into *S_s_* + *S_m_*. The momentum entropy, *S_m_*, is not affected by the physical process, so Δ*S* = Δ*S_s_*. (If the temperature *T* changes, then there is a contribution from *S_m_* as well, which can be computed analytically.) In practical applications, it is often preferable to compute spatial entropy in non-Cartesian coordinates, *Q*, but questions arise regarding the correct way to treat the entropy under a coordinate transformation because the differential Shannon entropy of information theory, which has the same mathematical form as the thermodynamic entropy, is not invariant under a change of coordinates. This lack of invariance appears problematic, because a simple change of coordinates must not affect the change in the entropy computed for a physical process.

This paradox is reconciled when one recognizes that the thermodynamic spatial entropy does in fact transform in the same manner as the differential Shannon entropy, but that the change in the transformed spatial entropy, Δ *S̃_s_*, is not in general equal to the change in total entropy, Δ*S*. The reason is that, in the new coordinates, unlike in Cartesians, the physical process also produces a change Δ *S̃_m_* in the entropy associated with the conjugate momenta, where *S̃_m_* is defined as an average of the momentum entropy over all values of the spatial coordinates. This change in the transformed momentum entropy precisely cancels the change in the spatial entropy associated with the transformation of coordinates, so that the change in total entropy due to the physical process is invariant under the transformation of coordinates.

The present analysis furthermore has provided useful expressions for the total entropy change for an isothermal physical process in terms of the spatial entropy in any set of spatial coordinates.
